# The Worldview Attitudes and Self-Determination in Russian Students who are Different Passion Degree of Tolkien Fans

**DOI:** 10.1192/j.eurpsy.2022.1762

**Published:** 2022-09-01

**Authors:** D. Zakrevskaya, T. Sadovnikova

**Affiliations:** Lomonosov Moscow State University, Faculty Of Psychology, Moscow, Russian Federation

**Keywords:** worldview, self-determination, escapism, psychological health

## Abstract

**Introduction:**

Self-determination is the developmental task (R. Havighurst) in youth leading to personality psychological well-being and health.

**Objectives:**

The aim was to study the worldview attitudes in Russian youth who are Tolkien fans.

**Methods:**

The techniques were completed by 121 students; 17-28 age (M = 21.7; M/F= 35.5 / 64.5 %); 81 (66.9%) subscribers of the Vkontakte communities dedicated to the work of J.R.R. Tolkien. 1. Incomplete Sentences by Sacks-Levy in authors’ version 2. World assumptions scale (R. Janoff-Bulman) (Padun, Kotelnikova, 2007) 3. Purpose-in-Life Test (J. Crumbaugh, L. Maholick) (Leontiev, 2000) 4. The Level of Escapism (Teslavskaya, Savchenko, 2019).

**Results:**

Three groups of respondents differing in their attitude to the literary genre of fantasy («fans», «amateurs», «indifferent») were identified. Respondents consider the world to be moderately benevolent, and fair, and themselves quite good people, able to control most of the events taking place in their lives. They are sure that they are often lucky in life. «Fans» have the highest tendency to escapism, the most pessimistic view of the world, with a more positive image of the person compared to other respondents. «Fans» consider their lives less emotionally rich and productive, compared to the assessment of their lives by peers of other groups.

**Conclusions:**

The level of escapism expression in fantasy literature fans are higher than in respondents who do not distinguish this genre as preferred. A new theoretical and empirical work is that worldviews can be monitored and taken into account in practical psychological working with at-risk young people.

**Disclosure:**

No significant relationships.

